# Emotion Variation from Controlling Contrast of Visual Contents through EEG-Based Deep Emotion Recognition

**DOI:** 10.3390/s20164543

**Published:** 2020-08-13

**Authors:** Heekyung Yang, Jongdae Han, Kyungha Min

**Affiliations:** 1 Division of Software Convergence, Sangmyung University, Seoul 03016, Korea; yanghk@smu.ac.kr; 2 Department of Computer Science, Sangmyung University, Seoul 03016, Korea

**Keywords:** emotion, EEG, Dataset for Emotion Analysis using Physiological Signals (DEAP), convolutional neural network (CNN), contrast, visual contents

## Abstract

Visual contents such as movies and animation evoke various human emotions. We examine an argument that the emotion from the visual contents may vary according to the contrast control of the scenes contained in the contents. We sample three emotions including positive, neutral and negative to prove our argument. We also sample several scenes of these emotions from visual contents and control the contrast of the scenes. We manipulate the contrast of the scenes and measure the change of valence and arousal from human participants who watch the contents using a deep emotion recognition module based on electroencephalography (EEG) signals. As a result, we conclude that the enhancement of contrast induces the increase of valence, while the reduction of contrast induces the decrease. Meanwhile, the contrast control affects arousal on a very minute scale.

## 1. Introduction

We watch and appreciate various visual contents such as movies, dramas, animation etc. The scenes from the contents evoke diverse emotional responses from us. The emotions from the scene can be categorized through Russell’s emotion model such as excitement, happy, pleased, peaceful, calm, gloomy, sad, fear and suspense [1]. The story line of visual contents is regarded as one of the most important factors that evoke a specific emotional response. For example, the battle scene between the fisher and sharks from an animation “The old man and the sea” evokes suspense. The scene of the settled forks from an animation “The man who plant trees” evokes peaceful or calm. We regard the story line of visual contents as latent factor of an emotional response, since it is the most important and major factor that affects the emotional responses. In addition to the story line, many researchers have questioned whether the representation of visual contents such as colors or contrasts can affect the emotional responses. Since color of a visual contents such as color of a cloth is very hard to change, we concentrate on the contrast of a scene, which affects the whole scene of the contents.

The purpose of our research is to examine how the emotional responses from visual contents are affected from the change of the contrast of a scene. Enhancing or reducing the contrast is expected to increase or decrease the emotional responses, respectively. In many applications, positive emotional responses such as happy or peaceful are magnified to delight the audiences further. Negative responses such as sad or fear are required to get reduced to relieve the audiences who feel uncomfortable for the scenes.

The assumption of this study that the emotional responses can be affected from the change of the contrast is backgrounded from biological observation. The lower or higher contrast can affect the number of photons arriving at the retina of an eye. Since the difference of the intensities of the photons is reduced, the brain reflecting on the neurons of retina can produce different biosignals, since the different biosignals can be interpreted as the change of valence and arousal. The relationship between the contrast and emotional responses should be examined in a very careful approach.

**Figure 1:**
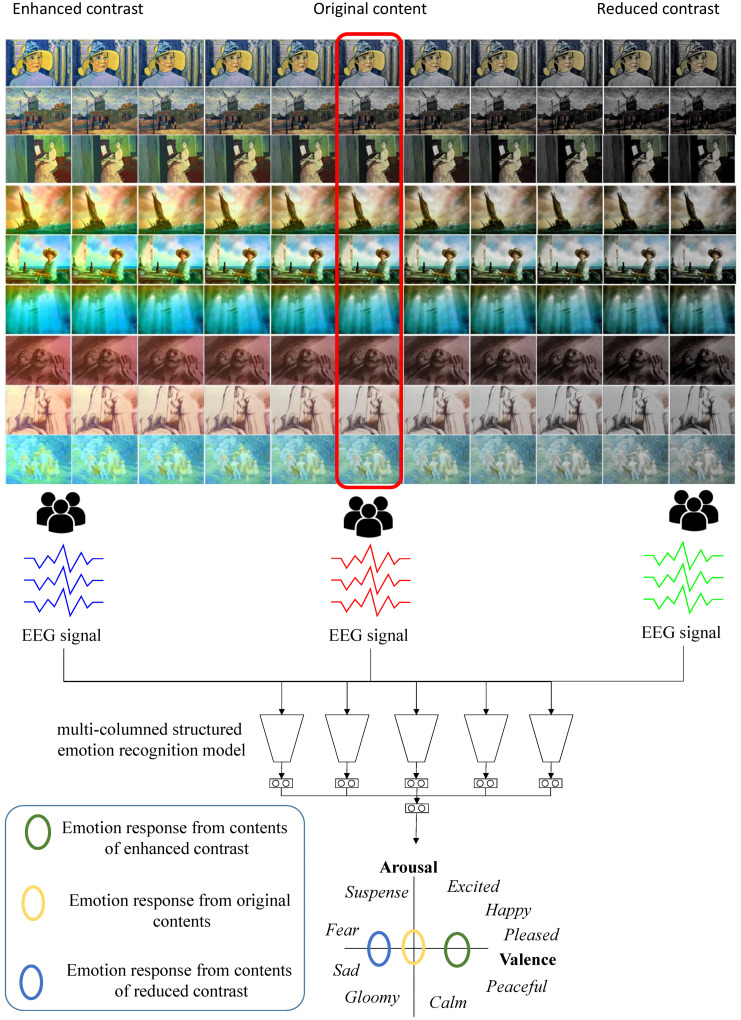
The overview of our framework: three groups of participants watch three different categories of contents: contrast enhanced, original, and contrast reduced. We process their EEG signals through our multi-columned emotion recognition model and estimate their valence and arousal.

To reveal the relationship between the contrast and emotional response, we collect some scenes from various visual contents of three representative emotions: neutral, positive and negative. Then, we control the contrast of the scenes in both directions, either enhancing or reducing. For each scene manipulated, we employ human participants to appreciate the contents, while their EEG biosignals are captured and processed using a deep learning-based emotion recognition model. We prove that the contrast change of a visual contents affect the arousal or valence estimated from the emotion recognition model. The overview of our framework is illustrated in Figure 1.

The estimation of the relations between contrast and valance/arousal by participants’ annotation is a subjective approach. For a reliable estimation of the relation, a series of objective approaches that employ the biosignals estimated from the participants. Many researchers employ diverse biosignals including EEG, electrocardiography (ECG), electromyography (EMG), photoplethysmogram (PPG), respiration rate (RSP) and galvanic skin response (GSR), etc. Since people cannot control their own biosignals, the biosignal-based approaches for estimating valence and arousal gather higher confidence than user survey-based approaches.

Furthermore, we carefully experiment the change of emotion recognition for the visual contents and compare the magnitude of emotion change due to the contrast control. From this experiment, we can suggest how to control visual contents to strengthen or weaken the emotion of the contents.

The change of emotion is expressed by the value of valence and arousal, which are measured through EEG biosignal. Actually, many researchers have presented a series of models that measure valence and arousal from EEG signal. Among these works, some of them employ classical machine learning schemes including Bayesian [2], dual tree-complex wavelet packet transform (DT-CWPT ) [3], decision fusion [4], support vector machine (SVM) [5,6], hidden Markov Model (HMM) [7] and brain computer interface (BCI) [8]. Some recent models employ deep learning schemes such as long short term memory (LSTM) recurrent neural network (RNN) [9], convolutional recurrent neural network (CRNN) [10], 3D CNN [11], LSTM [12] and multi-columned CNN [13]. The models using conventional machine learning schemes show about 70% accuracy, while the recent deep learning-based models show about 80% accuracy.

This paper is organized as follows. In Section 2, we briefly review several works for deep learning-based emotion recognition model and relationship between chroma and human emotion. In Section 3, we present outlines of our experiment including how sample movie clips are selected from visual contents and contrast of these clips are controlled. We explain our emotion recognition model and experiment strategy in Section 4, and present analysis and limitation of our work in Section 5. Finally, we draw conclusion of our work and suggest the direction of future work in Section 6.

## 2. Related Work

In this section, we describe previous studies around deep learning, emotion recognition approaches and emotional responses from visual contents.

### 2.1. Deep Learning-Based Emotion Recognition Models

#### 2.1.1. Early Models

Researchers employed handcrafted features to apply them to a variety of machine learning algorithms such as support vector machine (SVM) and principal component analysis (PCA). They showed acceptable accuracy, but the performance of their framework heavily relied on feature selection.

Many researchers have employed deep learning-based approach which does not require delicate feature selection process. Jirayucharoensak et al. [14] proposed a model of recognizing emotion from unstationary EEG signals. They used stacked autoencoder (SAE) to learn features from EEG signals. To minimize unstationary effect, they exploited PCA that extracts the most important component and covariate shift adaptation, which is also effective to avoid overfitting.

Khosrowabadi et al. [15] presented a multi-layer feedforward network model to distinguish emotion from unstationary EEG signals. The network model consists of spectral filtering layer for analyzing input signals and shift register memory layers. They identified emotion and employed arousal and valence level to represent the emotion according to Russell’s model.

#### 2.1.2. Deep Learning-Based Models

The early frameworks for emotion recognition have a common limitation in selecting features by human experts’ hands. As the progress of convolutional neural network (CNN), many researchers have overcome such a limitation. Salama et al. [11] presented 3D CNN model to recognize emotion from multi-channel structured EEG signals. Since EEG signals are spatio-temporal, their 3D CNN is a quite proper structure to be fed the signals. They also showed that data augmentation phase is helpful to improve the performance of emotion recognition and avoid overfitting. They tested the proposed model on the DEAP dataset and showed 88.49% and 87.44% accuracy with regard to arousal and valence, respectively.

Moon et al. [16] proposed a CNN-based model for recognizing emotion from EEG signals. Their main distinguishing point is connectivity features which is useful to describe synchronous activation in many different regions of brain. Therefore, they effectively acquired a variety of asymmetric brain activity patterns which is a key role to recognize emotion. They were not solely dependent on CNN structure, but showed that the partial feature selection contributes to improving the CNN-based approaches.

Croce et al. [17] classified brain and artifactual independent components by employing CNN-based model. Although, their research goal was not emotion recognition, but independent component analysis is essential to conventional feature selection approach. They conducted heuristic selection of machinery hyperparameters and CNN-based self-selection of the interesting features. They showed acceptable performance with cross validation 92.4% for EEG.

Yang et al. [13] proposed multi-channel structured CNN model to recognize emotion from unstationary EEG signals. Their model consisted of several independent recognizing modules, where they designed each module based on DenseNet [18]. For the final decision, they merged the results from the modules. They compared their performance with the existing studies and showed better accuracy for valence and arousal. They also extended their emotion recognizer to apply the task of distinguishing emotional responses to photographs and artwork [19].

Zhang et al. [20] presented an effective spatial attention map (SAM) for weighing multi-hierarchical convolutional features. SAM is useful for suppressing filter values corresponding to background features. They also proposed multi-model adaptive response fusion (MAF) approach which is for adaptive weighted fusion of multiple response maps generated by attentional feature.

Recurrent neural network (RNN) is widely used for processing time series data such as writing. Since EEG signals are time-sequential, many researchers have employed RNN to recognize emotion from the signals. Li et al. [21] proposed emotion recognizer based on RNN structure. They focused on three types of properties of EEG signals: frequency, spatial and temporal. To interpret the EEG signals in frequency and spatial domain, they made extraction of rational asymmetry (RASM). They extracted temporal correlation through LSTM RNN structure. They tested the proposed model by employing DEAP dataset and showed 76.67% mean accuracy.

Xing et al. [12] developed a model for recognizing emotion by employing multi-channel EEG. Their framework consists of an emotion time model and a linear EEG mixing model. The framework separate source of EEG signals from collected EEG signals, which improve the performance of classifying. The technical background of EEG mixing model is based on emotion timing model and stacked autoencoder (SAE). They tested their model using DEAP dataset and show the 81.10% and 74.38% accuracy for valence and arousal, respectively.

Several researchers employed conventional feature selection model to improve deep learning-based model. Some researchers often applied deep learning-based model to the other machine learning framework, other researchers used several different deep learning-based models.

Yoo et al. [22] presented a neural network-based recognizer for six types of emotional states including happy, joy, fear, anger, sadness and despair. They tested their proposed model for a variety of multi-modal bio-signals including EEG, PPG, GSR and ECG.

Yang et al. [23] proposed hybrid neural network-based model by combining RNN and CNN for recognizing emotion. They employed baseline signals in pre-processing phase, which improved the performance of recognition. Such pre-processing method resembles conventional feature-based techniques. They also had the proposed model to learn the way of representing unstationary EEG signals for recognizing emotion. In their hybrid model, the RNN module makes an extraction of contextual information from signals and the CNN module measures inter-channel correlations among EEG signals.

#### 2.1.3. Relationship Between Chroma and Human Emotion

In early days, Adams and Osgood [24] presented a classic research on the affective meaning of color, which pioneers to reveal the relationship between color and affections.

Recently, Suk et al. [25] researched characteristics of emotional responses to hue, lightness and chroma. In this study, subjects are asked for assessing their emotional response against various color stimulus. The result shows chroma level and lightness significantly affect valence and arousal. Although the result support that color stimulus could incur emotional response, the study relies on subjective self assessment whereas our research utilizes objective EEG signal.

Jun et al. [26] performed a study to find out how can we modify an image’s color characteristics in order to enhance its emotional impact. Measurements are performed to how emotional responses are changed due to different color and contrast, also regarding contexts of the image. They make use of skin conductance and heartbeat rate variation to quantify emotion. In the result, color and contrast clearly affect emotional response, but aspect of the emotional impact is different amongst context of the image. The study confirms our priori that emotional response is affected by colors and gives future study improvements.

Rajae-Joordens [27] insisted the effect of hue, lightness and saturation of colored light upon human arousal and valence is significant. In the research, both objective and subjective measurements for emotional response under various colored lights are performed. Although the result proves features of color seem to affect human emotion, discrepancy between results from objective and subjective measurements also explains why there are different aspect of change in emotional responses between various studies.

With recent advances in deep learning, various image transfer techniques are introduced. Peng et al. [28] studied how emotional responses are being changed by transferring image to another. In general, image transfer techniques lead in changed color tone and texture. A quantitative evaluation is performed to find out differences in emotions from against original image to image transferred one. The result shows image transference, in terms of color and texture, evokes some change in emotional response, albeit causal analysis against color or contrast are not discussed.

## 3. Contrast Change of Visual Contents

### 3.1. Sampling Clips of Three Representative Emotions

We selected three well-known animations for our study: *Loving Vincent* [29], *The old man and the sea* [30], and *A man who plants trees* [31]. These animations are recognized as distinctive rendering styles. We selected the artistically styled contents, since we concentrated on the color variation of the contents. Since the color variation of artistic styled image was narrower than that of real photograph, the effect of contrast control could be more drastic for artistic styled images. Therefore, the effect of contrast control on artistically styled images for emotion variation could be more dominant than that on real photographs. We will extend the target visual contents to real photographs in future work.

**Figure 2:**
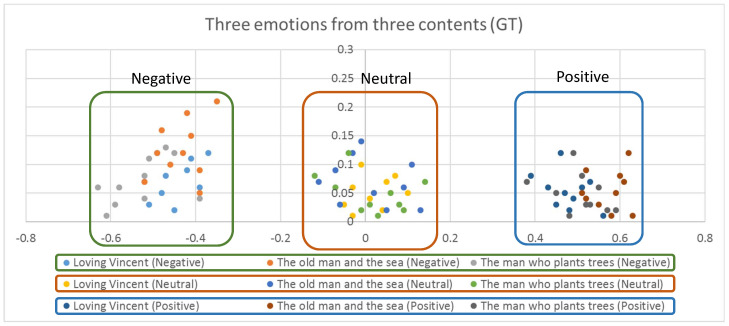
Ground truth emotions estimated from the clips sampled from the three contents.

From the selected visual contents, we sampled three different scenes, each of which contain positive, neutral and negative emotion, respectively. For this purpose, we sampled 10 scenes from each of the contents and hired 10 human participants to mark the emotion of the scene for the three categories: positive, neural, and negative. We also asked the participants to suggest the background of their selection. From the votes of the participants, we selected the scenes of highest votes for our experiment. The emotions from the sampled clips for three emotions are illustrated in Figure 2, which were estimated from the model we describe in Section 4. The emotions from the clips were grouped into three different emotions: positive, neutral and negative. From Figure 2, we concluded that the emotions marked by 10 human participants on the clips were correct.

#### 3.1.1. Scenes of Positive Emotion

From *Loving Vincent*, the scene where Armand talks to Marguerite, the daughter of Dr. Gachet, about good memories of Vincent van Gogh was selected as the scene of most positive emotion. The major reason of the selection is that this scene that reminds of a positive memory about Vincent evokes positive emotion.

From *The old man and the sea*, the scene of the fantasy that a boy is playing with fishes in the sea was selected. The major reason of the selection is that playing with fishes in the sea is a fantasy from childhoods.

From *A man who plants trees*, the scene of happy people living in a village filled with a lot of trees was selected. The major reason is that this last scene of the animation shows how the effort of a man can make people happy and a village flourish.

**Figure 3:**
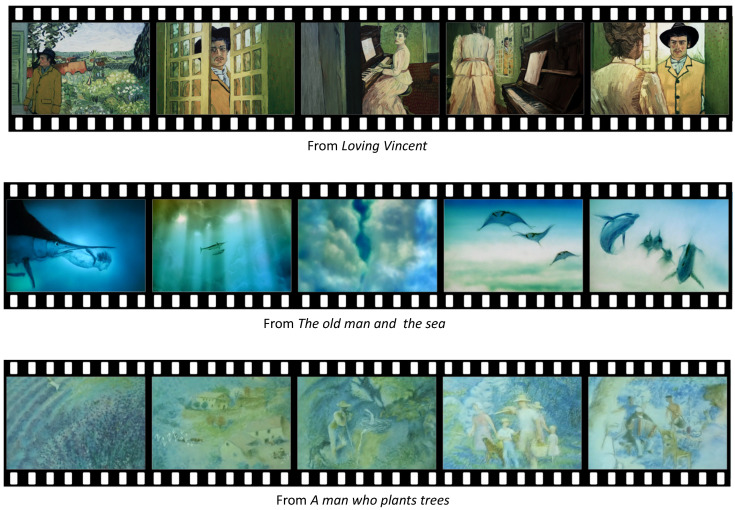
The scenes of positive emotion.

The scenes of positive emotion are illustrated in Figure 3.

#### 3.1.2. Scenes of Neutral Emotion

Most of the participants selected the scenes that happened early in the contents as scenes of neutral emotion. Since the early scenes introduced the actors of the contents and explain their situations, the emotional response from these scenes tended to be neutral.

From *Loving Vincent*, the scene where Armand is visiting Auvers was selected. They selected this scene since visiting Auvers is an action that does not evoke any emotion responses.

From *The old man and the sea*, the opening scene where a boy visits the old man and talks about his dream was selected. Similar to the *Loving Vincent* scene, talking about a dream does not evoke either positive or negative emotions.

From *A man who plants trees*, the scene where the narrator encounters Elzeard Bouffier and visits his house was selected. Similar to the above contents, visiting a house does not evoke emotional responses.

**Figure 4:**
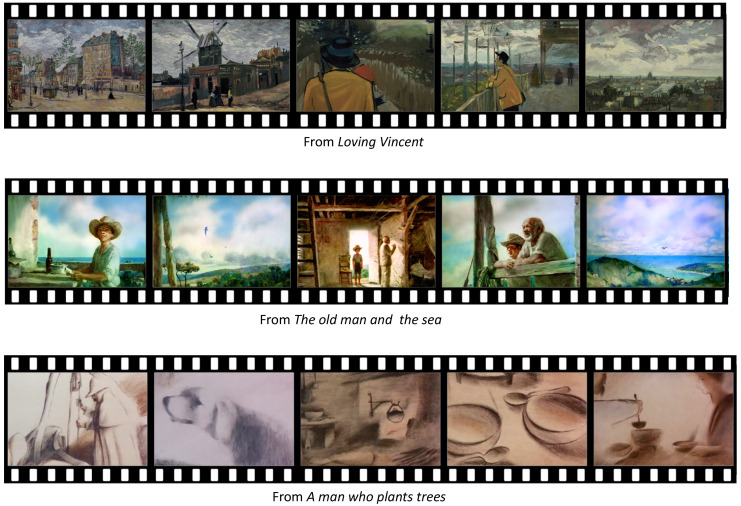
The scenes of neutral emotion.

The scenes of neutral emotion are illustrated in Figure 4.

#### 3.1.3. Scenes of Negative Emotion

Most of the participants selected scenes of conflict as the scenes of negative emotion. The existence of unpleasant objects such as blood accelerated the negative emotion.

From *Loving Vincent*, the scene where Louise Chevalier, the maid of Dr. Gachet speaks ill of Vincent van Gogh was selected as a scene of negative emotion. Since most of the participants had good feelings about Vincent, they felt unpleasant and negative about the conversation between Louise and Armand.

From *The old man and the sea*, the scene of bloody struggle between sharks that came to hunt the tuna caught by the old man was selected as negative. The blood spread on the sea added the negative emotion.

From *A man who plants trees*, the scene that describes the process of falling down in a village due to the harsh weather was selected as a scene of negative emotion.

**Figure 5:**
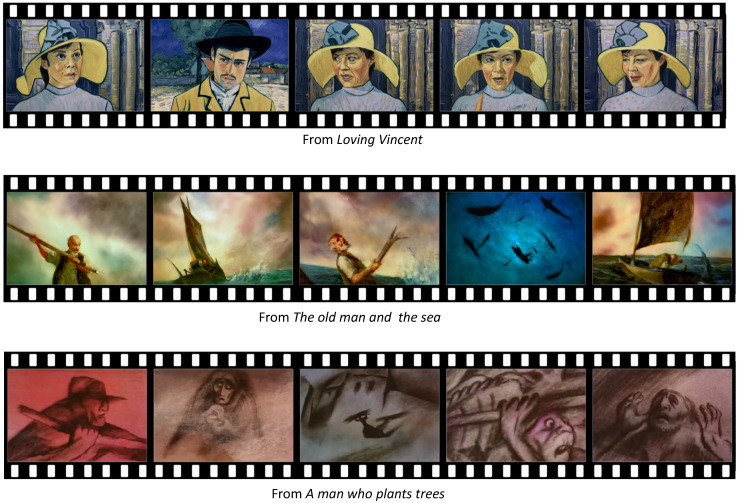
The scenes of negative emotion.

The scenes of negative emotion are illustrated in Figure 5.

### 3.2. Controlling the Contrast of the Scenes

We applied linear contrast control scheme, which has been widely used in many image processing applications. The reason is that both contrast enhancement and contrast reduction were required. Since we did not want to affect color factor in the contrast control, we converted the color in RGB space into HSV space and applied contrast control for the S (saturation) and V (value). After the control, we re-converted the color in HSV space into RGB space.

A contrast controlled value of saturation, denoted as $S^{\prime }$, was estimated as follows:

$$S^{\prime }=S+\left(S-S_{avg}\right)*\alpha ,$$

where $S_{avg}$ is the average value of the saturation, and α is a control parameter. We set α as a negative value for contrast reduction. We control the contrast of the scenes from the selected visual contents. The result of the control is shown in Appendix A. We set α=0.1 for enhancement and α=−0.1 for reduction.

## 4. Implementation and Results

### 4.1. Implementation Detail

**Figure 6:**
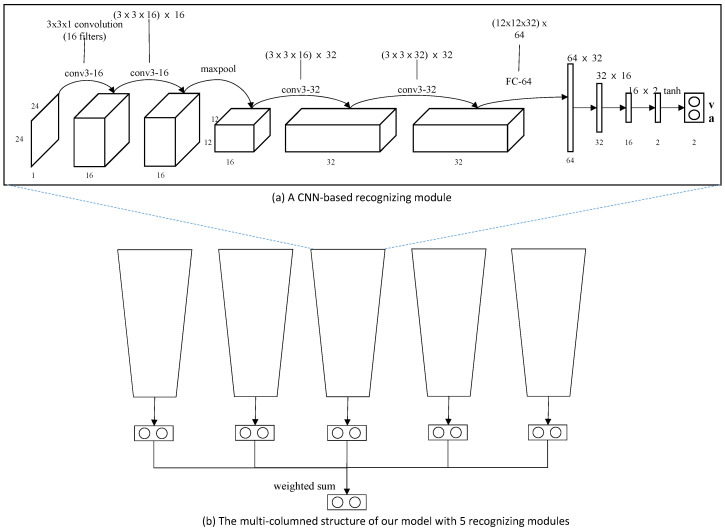
Our multi-columned emotion recognition model with five recognizing modules: Each module is composed of four convolutional layers, one max pooling layer and four fully connected layers. The activation function we employ is a tanh function, which produces values in (−1,∼1). The result of each module is a paired value of valence and arousal.

We implemented our model in a personal computer with Intel Core i7 9600 CPU (Intel, San Jose, CA, USA), 16 GB main memory, and nVidia GeForce TitanX GPU (nVidia, Santa Clara, CA, USA). The model is implemented using Python with Pytorch library. Our multi-column structured emotion recognition model was derived from our previous research [13]. The structure of our emotion recognition model is illustrated in Figure 6. In this work, we build our recognition model with five recognizing modules, since Yang et al. revealed that five modules showed the best performance in recognizing emotion.

Hence the model was trained with a DEAP dataset [32], we provided a similar setup with the DEAP. We used LiveAmp 32 and LiveCap [33], which allowed us to set up 32 channels following a standard 10/20 system [34].

### 4.2. Data Collection

**Figure 7:**
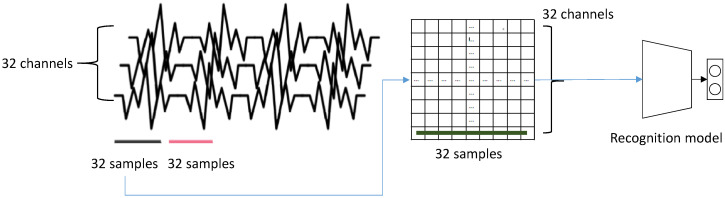
The construction of input data: 32 consecutive samples captured from 32 EEG channels constructs a single input vector for a recognizing module. Since we use five modules in our recognition model, five consecutive samples are captured.

We followed the data collection strategy in [13]. For a training, we downsampled the EEG signals of DEAP dataset to 128 Hz and applied a 4.0–4.5 Hz band pass filter. As a result, we sampled 128×60 samples from each trial for 40 channels. Among them, we excluded eight channels for the normalization. 32 channels of EEG signal became one row of our 2D input. Therefore, we sampled 32 consecutive samples from a single input for our model. Figure 7 illustrates the construction of a single input data. For a test, we similarly built 32×32 input data using the EEG signals captured from participants.

### 4.3. Deep Emotion Recognition Model

As we described before, three scenes were selected from each animation. We assumed that contrast would more or less affects emotional response by the mood of the scene. 30 participants were asked to watch each of the positive, neutral, and negative scenes from an animation, then move to next animation after 30 s of rest. After nine playbacks, another nine playbacks were followed with increased contrast, then last nine with decreased contrast. Each playthrough, consisting of 27 playbacks, was preceded by 3 s of baseline recording. All recordings were started/stopped by human control, allowing less than 1 s of error.

### 4.4. Model Training

To streamline our data to DEAP dataset, similar preprocessing is taken. We used EEGLab with MATLAB to preprocess those recordings alongside the channel location file. The data were downsampled to 128 Hz with a bandpass frequency filter from 4.0–45.0 Hz was applied. The order of the channels was same as that of DEAP. The data were segmented into 27 trials. Our emotion recognition model got preprocessed data and produced estimation upon valence and arousal level for each trial.

Our model was trained using the DEAP dataset according to the strategy presented by [13]. For training and validation, DEAP dataset, which was composed of the EEG signals captured from 32 participants, was segmented into three subsets: 22 participants for training, five for validation, and five for testing. Since each participant executed 40 experiments, the experiment data in the datasets for training, validation and test were 880, 200, and 200, respectively. Furthermore, we sampled 32 consecutive values from equally-spaced different positions of an experiment data whose distance was 7680/(32∗k). Note that *k* is the number of modules of our model. We set *k* as 5 in this study. In total, the number of training, validation and test samples for our model was 42,240, 9600 and 9600.

In many related literatures, various models have been presented to estimate valence and arousal from EEG biosignal. They employ either classic machine learning techniques [2,3,4,5,6,7,8] or deep learning techniques [9,10,11,12,13]. According to [13], the machine learning technique-based schemes present 71.66% accuracy for valence and 69.37% for arousal in average, and the deep learning-based schemes present 81.4% and 80.5%, respectively. These research trends present a strong background for us to employ a deep learning-based model for estimating valence and arousal using the EEG signals captured from users. We present the precision, recall and F1 score of our model in Table 1, which was estimated in [13].

### 4.5. Experiment

**Figure 8:**
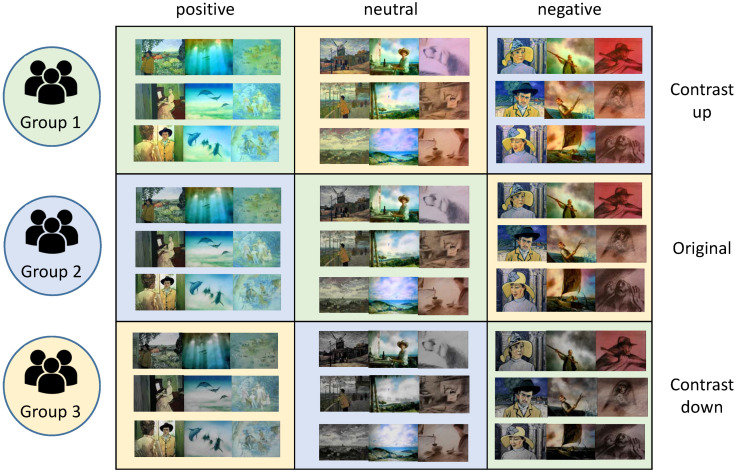
Nine categories of movie clips combined by contents of three emotions and three contrast variation. The color of each category corresponds to the human participant group.

We hired 30 human participants for our experiments and group them into three groups. The distribution of gender and age of the participants for each group are suggested in Table 2. Each group was required to watch three movie clips. We organized the movie clips into nine categories as illustrated in Figure 8. The groups represented by three colors such as green, blue and yellow were assigned to movie clips of different emotions.

**Figure 9:**
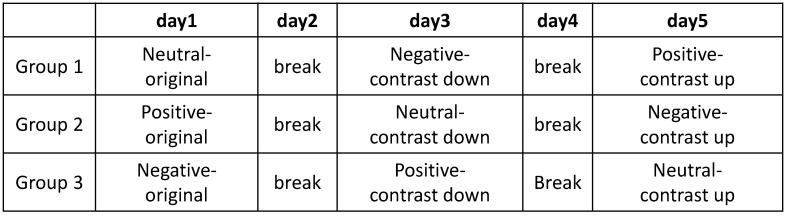
The schedule for experiment of three groups.

A participant of a group was guided to watch three movie clips that belong to the same category. The order of three movie clips was shuffled randomly. Each clip was played for 60∼90 s. After watching three movie clips that belonged to one category, the participants had a day break to neutralize their emotional responses. The schedule of our experiment is illustrated in Figure 9.

**Figure 10:**
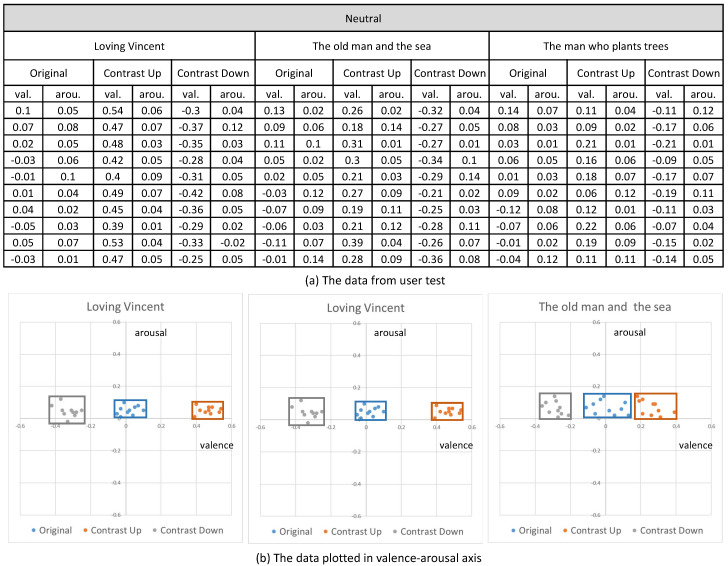
The valence and arousal extracted from the visual contents of neutral emotion.

**Figure 11:**
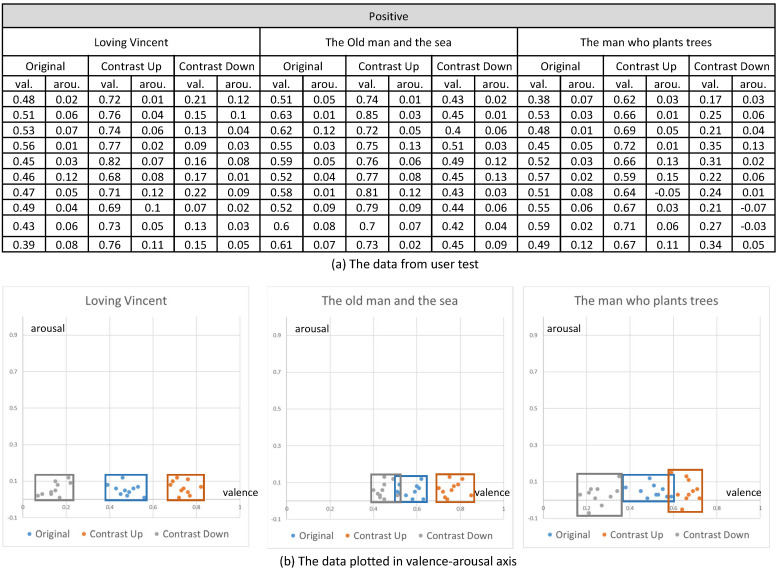
The valence and arousal extracted from the visual contents of positive emotion.

**Figure 12:**
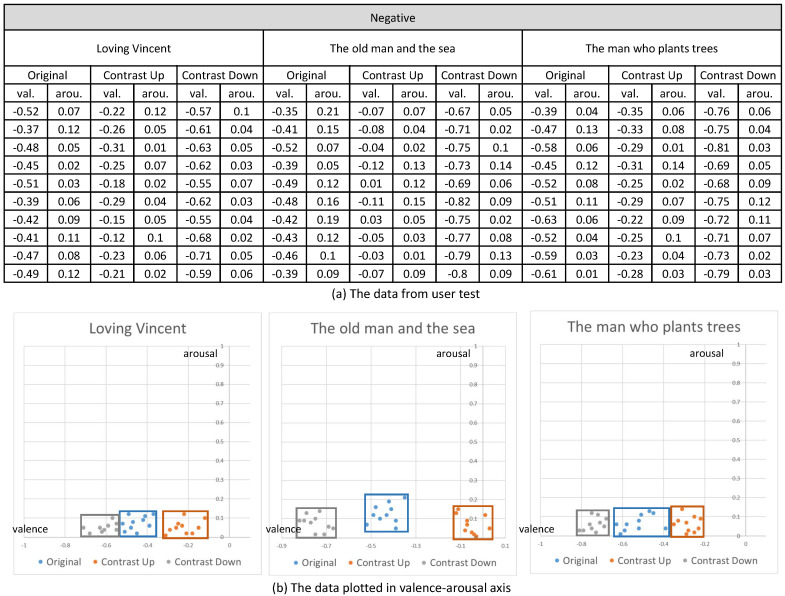
The valence and arousal extracted from the visual contents of negative emotion.

Their emotional responses expressed by their EEG signals were processed through our multi-stage emotion recognition module to extract valence and arousal. We present the valence and arousal extracted from our experiment in Figure 10, Figure 11 and Figure 12, respectively.

## 5. Analysis

### 5.1. Analysis 1: The Comparison of Groundtruth Emotions

In the first analysis, we compared the valence and arousal of the original three emotions suggested in Figure 10, Figure 11 and Figure 12 through *t*-test. As illustrated in Table 3, *p* values for the valence of the pair of neutral and negative and neutral and positive from the three contents were less than 0.01, which denotes that the difference of valence was significant. Meanwhile, *p* values for the arousals were all greater than 0.01, which denotes that the difference of valence was not significant. From the *t*-test, we concluded that the movie clips we selected from the three contents showed distinctive valence and similar arousal.

### 5.2. Analysis 2: The Difference of Emotions from the Control of Contrast Affects Valence

**Figure 13:**
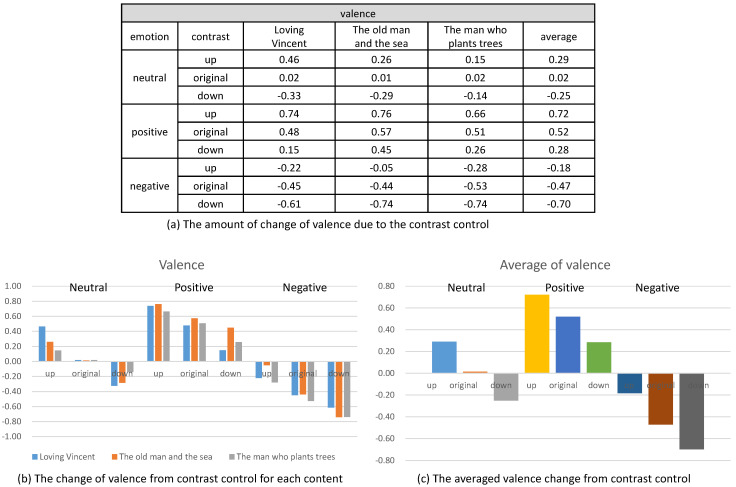
The change of valence from controlling contrast.

In the second analysis, we compared the change of valence and arousal due to the control of contrast. As illustrated in Figure 13, we recognized that the enhancement of contrast (up) increased the valence of the contents and the reduction of contrast (down) decreased the valence for all the three status of the emotion (neutral, positive and negative). Even though the amount of increase or decrease of the valence varied according to the content, the increase or decrease happened for all the contents.

**Figure 14:**
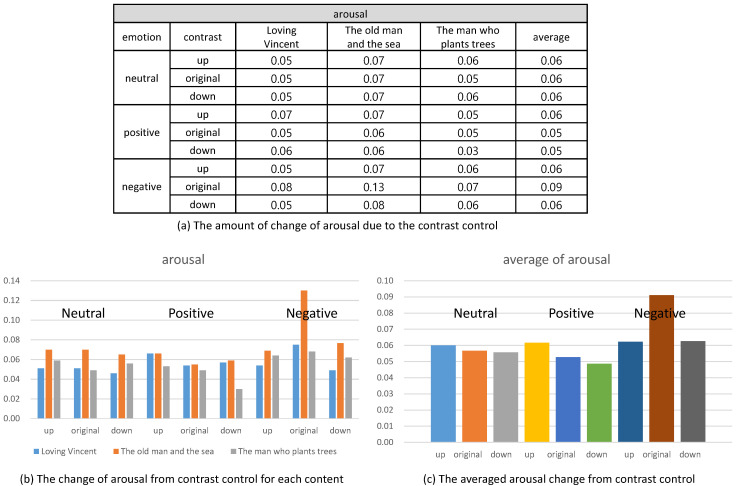
The change of arousal from controlling contrast.

On the contrary, the arousal did not show a specific change from the control of contrast. As illustrated in Figure 14, the control of contrast produced very minute change of arousal. Therefore, we conclude that the control of contrast affected the valence only. Furthermore, the enhancement of contrast increased valence and the reduction decreased valence. We executed *t*-test for the valence and arousal suggested in Figure 13 and Figure 14 and demonstrated that the valence was different significantly and that the arousal was similar. The result of the *t*-test is suggested in Table 4.

### 5.3. Analysis 3: Comparison of Enhanced Emotions to the Original Emotions

**Figure 15:**
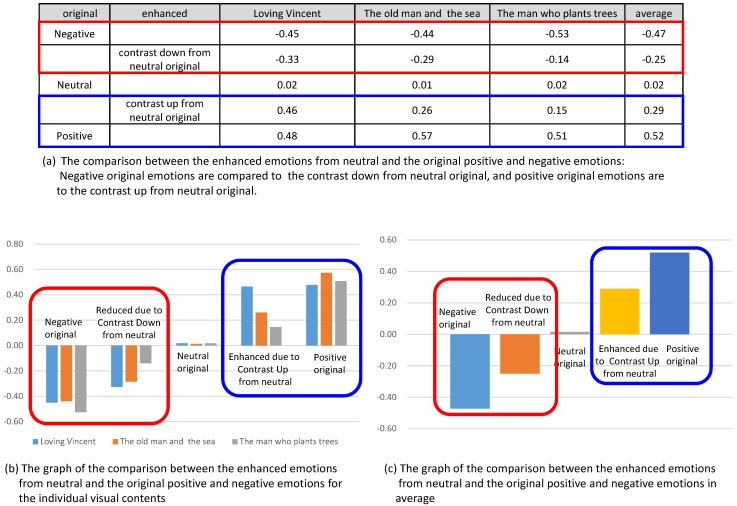
Comparison of the enhanced emotions from neutral and the original positive and negative emotions.

In this analysis, we compared the enhanced valence from the control of contrast to the valence of original contents. In Figure 15, the enhanced valence and reduced valence from the contents of originally neutral state were compared with the valence from the original positive and the original negative, respectively. Figure 15b shows the comparison for three contents and Figure 15c shows the comparison for the averaged valence from the three contents. In the graphs, we concluded that the enhanced or reduced amount of the valence did not reach the valence of original contents. For example, the valence from the originally neutral emotion was enhanced due to the contrast up, while the amount of the increased valence did not reach the valence of originally positive emotion.

**Figure 16:**
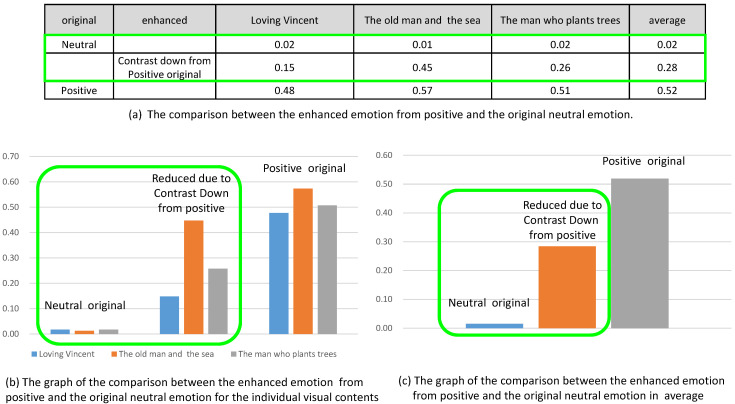
Comparison of the enhanced emotions from positive and the original neutral emotion.

The same result was observed for the originally positive and negative emotion. In Figure 16, we compared the reduced emotion due to contrast down from originally positive emotion to the neutral emotion. Even though the valence was reduced from the positive emotion, the valence was still greater than the valence of neutral emotion.

**Figure 17:**
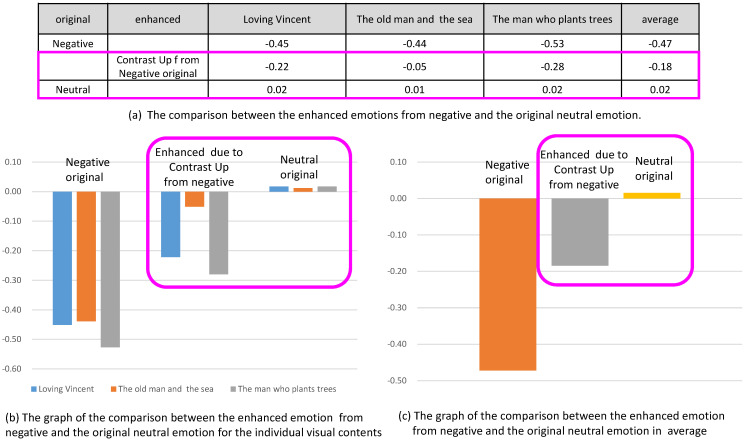
Comparison of the enhanced emotions from negative and the original neutral emotion.

Similarly, in Figure 17, the emotion due to contrast up from originally negative emotion was enhanced, which was recognized in the increase of valence. In this case, the valence enhanced from the negative emotion was still less than the valence of neutral emotion.

### 5.4. Analysis 4: Mostly Changed and Leastly Changed Contents

**Figure 18:**
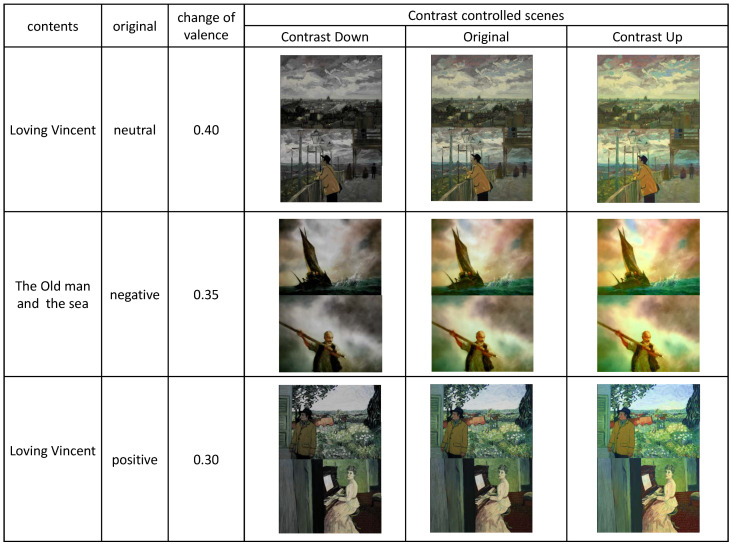
The contents whose emotions are mostly changed.

**Figure 19:**
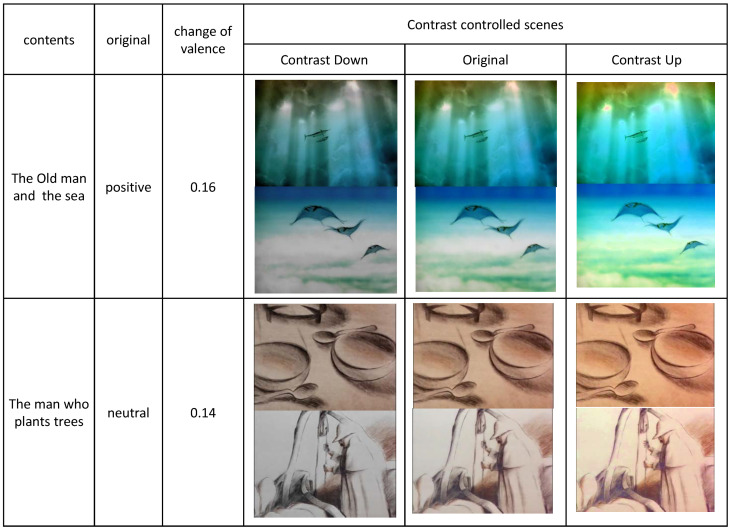
The contents whose emotions are leastly changed.

We compared the contents whose valence changed in the greatest scale in Figure 18 and in the least scale in Figure 19. In Figure 18, we present three scenes whose emotions changed in the greatest scale. As visually recognized, these contents contained landscape scenes with natural lights. Since these scenes contained many components of various color and tone, the increase of the contrast enriched the colorful expression of the scene, which enhanced the valence of the original contents. The decrease of the contrast, on the contrary, blunted the colorful expression of the scene, which reduced the valence of the original contents.

The scenes in Figure 19 were affected in the least scale from the control of contrast. Since these scenes were either fantastically illuminated scenes or indoor scenes, the colors in the components of the scenes were not diverse. These monotonous colors were less affected by the control of contrast, which resulted in the least enhanced or reduced valences from the original emotions.

### 5.5. Analysis 5: Existing Study

Since most other works considered mono-color as stimuli, it was hard to directly compare our result with other models. Jun et al. [26] pointed out that contents and contextual information may affect emotional response alongside with color itself, therefore we carefully chose certain scenes with emotional backgrounds. These scenes contained various colors and context, then raised leads in mixed emotional response. Our model was to make “scene-wise” change in contrast, rather than changing specific colors.

### 5.6. Limitation

**Figure 20:**
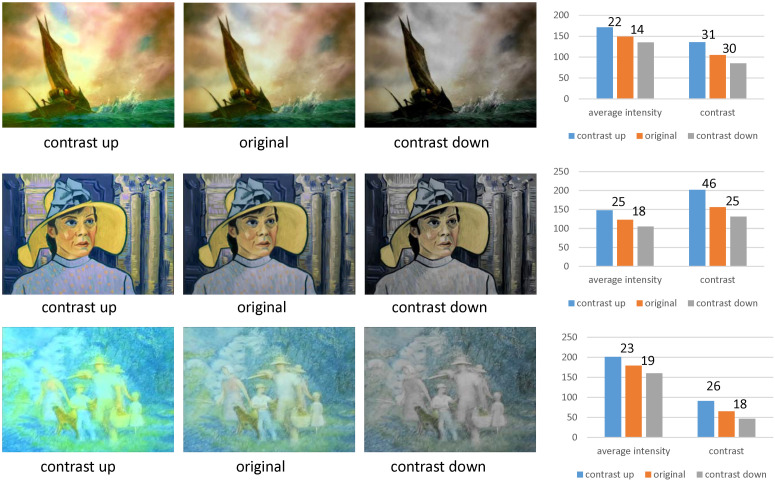
The change of contrast and intensity according to the contrast control.

An unexpected result of our contrast control scheme is that the intensity of the image also varies. Enhancing the contrast may increase the average intensity of the image and reducing may reduce the average intensity. In Figure 20, we sampled an image from three contents and estimate the change of contrast and average intensity. The change of contrast was measured by sorting the pixels according to their intensities and subtracting the average intensity of highest 10% pixels from that of darkest 10% pixels. The results are illustrated in the rightmost column of Figure 20.

Even though the average intensity of an image varied, we still argued that the increase or decrease of valence comes from the change of contrast rather than the average intensity in the following two points. The first point is that the magnitude of the change of contrast is greater than the magnitude of the change of average intensity. Since the change of contrast shows greater magnitudes than the change of average intensity, we argued that the change of valence is affected by the change of contrast rather than the change of average intensity.

Our second point is from existing literatures. Even though brightness is known to one of the cause of emotional response, the study of Wilms and Oberfeld [35] indicates that brightness is relatively weak driver of the change of emotional response. It causes limited increase for arousal and valence only for zero to lowly saturated colors. Therefore, we could assume that brightness is not the major driver of observed effect.

Our third point is to employ user-annotated valence-arousal values. Considering user-annotated valence-arousal values with the valence-arousal values estimated from EEG signals through emotion recognition model can improve the confidence on the performance of our approach. However, our experiment on participants collected only their EEG signals. Our next experiment on estimating human emotion using EEG signals will collect user annotated valence-arousal values as well as EEG biosignals and compare them to improve the confidence of our experiment.

## 6. Conclusions and Future Work

In this paper, we have proved that the contrast control of visual contents affect the valence of the people who watch the contents. The enhancement of contrast increases valence, while the reduction decreases. Arousal is not affected by the change of contrast. We have proved our argument by extracting EEG biosignals from human participants and by recognizing valence and arousal using a deep emotion recognition model.

We have a plan to extend this study to review the relations between other properties of contents such as color with the emotion of visual contents. We also have a plan to apply the result of this study in manipulating visual contents to satisfy users.

## Figures and Tables

**Table 1:** The precision, recall and F1 score.

Precision (%)		Recall (%)		F1 Score (%)	
Valence	Arousal	Valence	Arousal	Valence	Arousal
85.57	86.66	80.18	81.02	82.79	83.75

**Table 2:** Gender and age distribution of the participants.

	*Gender*		*Age*			
	Female	Male	<20	20s	30s	>40
group 1	6	4	2	7	1	0
group 2	5	5	1	7	1	0
group 3	5	5	1	8	1	0

**Table 3:** *p* values for the valences and arousals between neutral, positive and negative emotions from the three contents.

	*Loving Vincent*		*The Old Man and the Sea*		*The Man Who Plants Trees*	
	val.	arou.	val.	arou.	val.	arou.
Neutral and Positive	$3.84936\times 10^{-14}$	0.82521	$2.09928\times 10^{-11}$	0.39830	$7.92079\times 10^{-12}$	1.0
Neutral and Negative	$4.30645\times 10^{-14}$	0.10998	$2.28149\times 10^{-11}$	0.01567	$6.66321\times 10^{-12}$	0.27336

**Table 4:** *p* values for the valences and arousals between original contents and contrast controlled contents for three original emotion.

Original	Emotion	*Loving Vincent*		*The Old Man and the Sea*		*The Man Who Plants Trees*	
Emotions	Control	val.	arou.	val.	arou.	val.	arou.
**neutral**	original & up	$7.766\times 10^{-14}$	1.0	$5.879\times 10^{-7}$	1.0	$5.342\times 10^{-4}$	0.5527
	original & down	$6.859\times 10^{-12}$	0.7343	$7.803\times 10^{-9}$	0.7941	$3.451\times 10^{-5}$	0.6606
**positive**	original & up	$1.754\times 10^{-10}$	0.4495	$2.194\times 10^{-8}$	0.5248	$1.754\times 10^{-10}$	0.8598
	original & down	$9.166\times 10^{-12}$	0.8502	$1.025\times 10^{-6}$	0.8198	$9.166\times 10^{-12}$	0.3604
**negative**	original & up	$3.296\times 10^{-8}$	0.2025	$1.695\times 10^{-12}$	0.2526	$3.773\times 10^{-7}$	0.8279
	original & down	$1.618\times 10^{-6}$	0.0716	$4.981\times 10^{-11}$	0.0332	$2.108\times 10^{-6}$	0.7278

**Figure A1:**
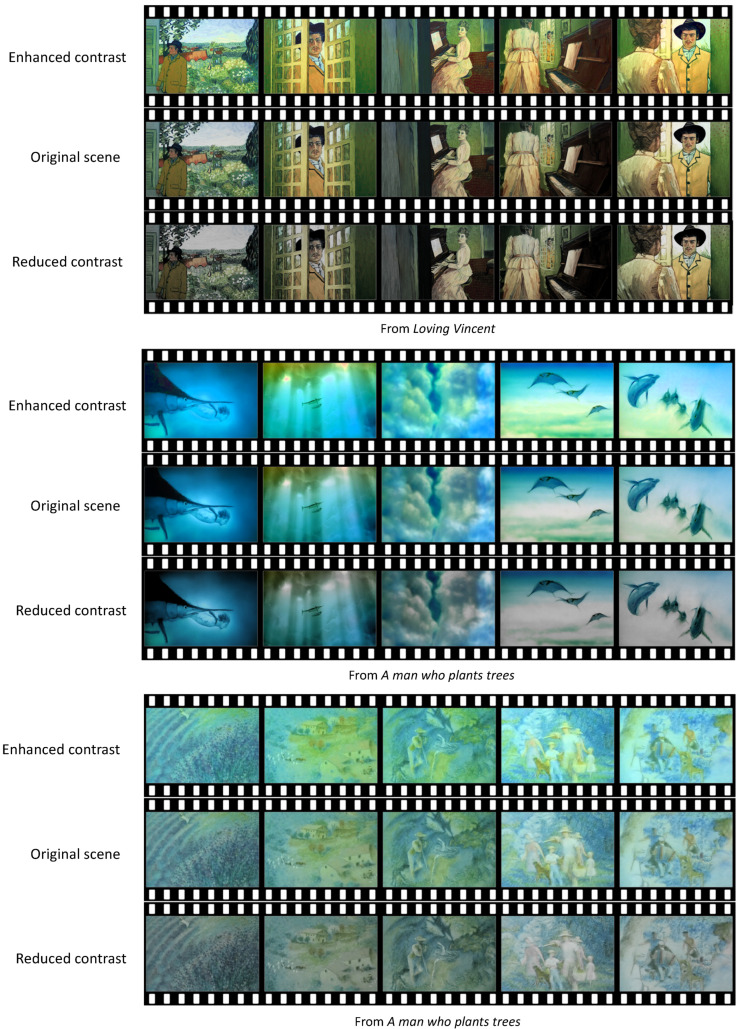
The contrast-controlled positive scenes.

**Figure A2:**
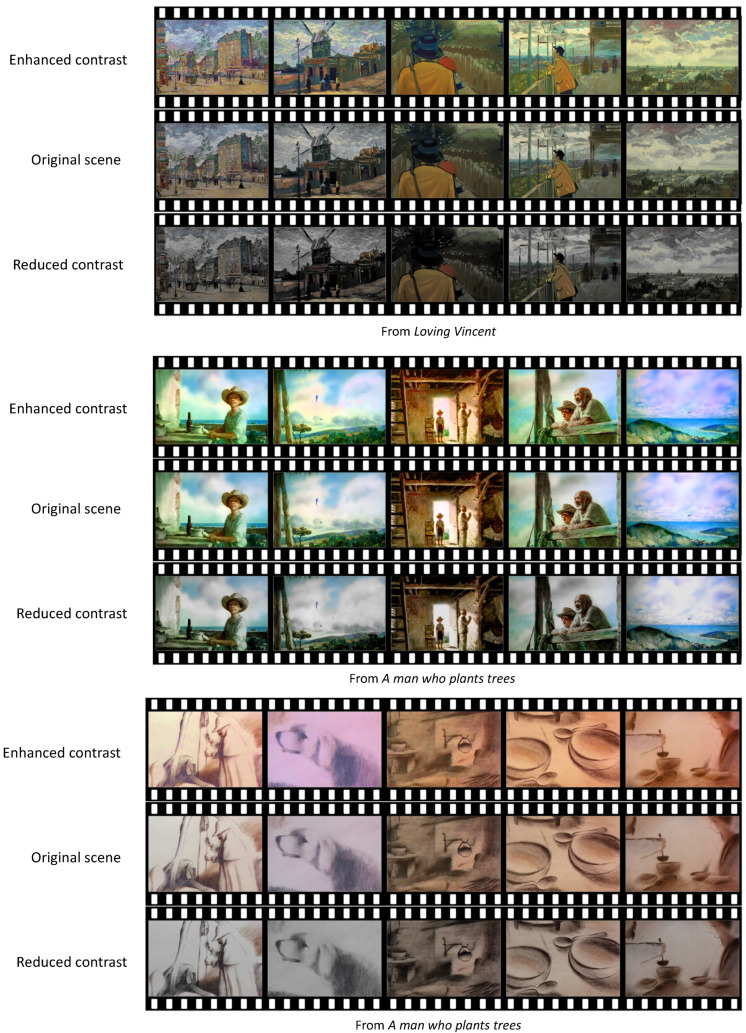
The contrast-controlled neutral scenes.

**Figure A3:**
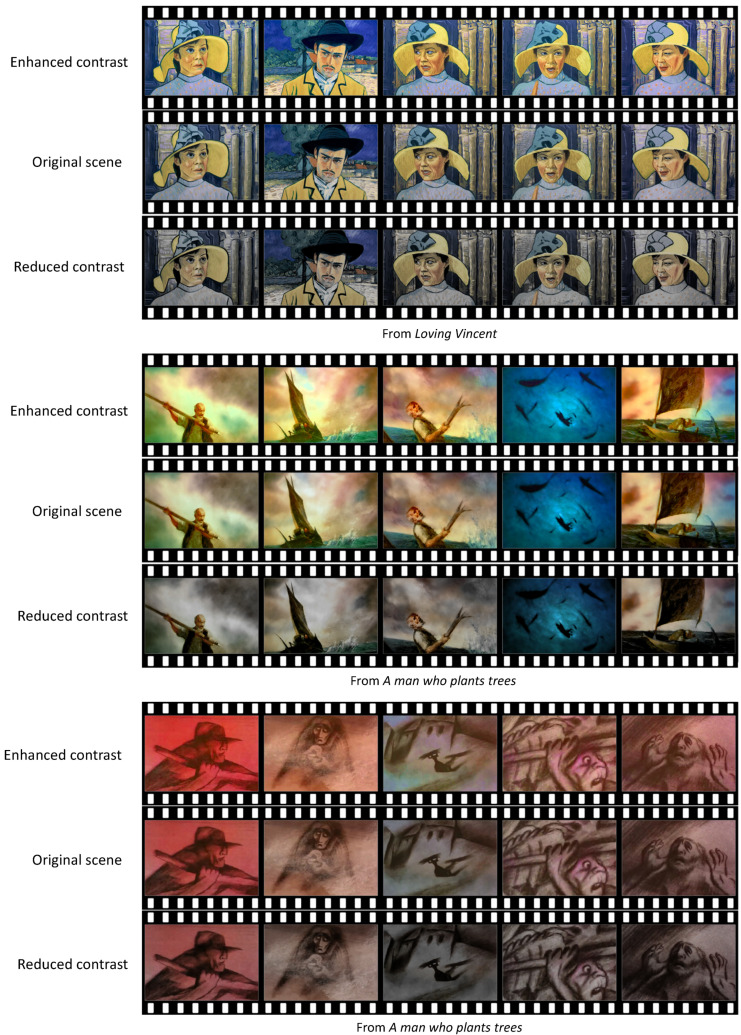
The contrast-controlled negative scenes.

## Appendix A

The contrast-controlled scenes from the selected visual contents.
